# Gulf Coast Ticks (*Amblyomma maculatum*) and *Rickettsia parkeri,* United States

**DOI:** 10.3201/eid1305.061468

**Published:** 2007-05

**Authors:** John W. Sumner, Lance A. Durden, Jerome Goddard, Ellen Y. Stromdahl, Kerry L. Clark, Will K. Reeves, Christopher D. Paddock

**Affiliations:** * Centers for Disease Control and Prevention, Atlanta, GA, USA; † Georgia Southern University, Statesboro, GA, USA; ‡ Mississippi Department of Health, Jackson, Mississippi, USA; § US Army Center for Health Promotion and Preventive Medicine, Aberdeen Proving Ground, Maryland, USA; ¶ University of North Florida, Jacksonville, Florida, USA

**Keywords:** Rickettsia parkeri, Amblyomma maculatum, spotted fever rickettsia, dispatch

## Abstract

Geographic distribution of *Rickettsia parkeri* in its US tick vector, *Amblyomma maculatum*, was evaluated by PCR. *R. parkeri* was detected in ticks from Florida, Georgia, Kentucky, Mississippi, Oklahoma, and South Carolina, which suggests that *A. maculatum* may be responsible for additional cases of *R. parkeri* rickettsiosis throughout much of its US range.

The Gulf Coast tick, *Amblyomma maculatum* (Figure), is a Nearctic and Neotropical hard tick found in coastal areas of the southern United States, with inland range extensions in Kansas, Oklahoma, and some other states. It is also found in regions of several Central and South American countries that border the Gulf of Mexico and Caribbean Sea, including Mexico, Guatemala, Belize, Nicaragua, Honduras, Costa Rica, Colombia, Venezuela, and some parts of Ecuador and Peru (*1*).

**Figure:**
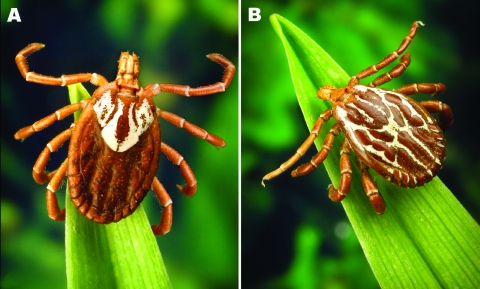
Adult *Amblyomma maculatum* (the Gulf Coast tick). A) Female; B) Male. Photographs courtesy of James Gathany, Centers for Disease Control and Prevention

*Rickettsia parkeri,* a member of the spotted fever group rickettsiae, was initially identified in Gulf Coast ticks in 1937(*2*)*.* In 2004, the first confirmed human infection with *R. parkeri* was reported (*3*). Since that report, confirmed cases of *R. parkeri* rickettsiosis have been identified in other persons in Mississippi, Virginia, and possibly other US states (*4**–**6*). Only a few studies, each conducted >50 years ago, document the occurrence of *R. parkeri* in *A. maculatum* ticks (*2**,**7**,**8*). No contemporary surveys have documented the range of *R. parkeri* in the United States or the frequency of *R. parkeri* infection in collections of individual Gulf Coast ticks.

## The Study

*A. macu*latum ticks collected during 1996–2005 were evaluated by molecular methods for evidence of infection with *R. parkeri*. Ticks were collected from various locations in Florida, Georgia, Kentucky, Mississippi, Oklahoma, and South Carolina. Most were questing adults collected from vegetation by using flannel cloth flags; a few crawling, nonattached, nonengorged adults were obtained (4 from a coyote and 3 from human hosts), and 1 engorged nymph was removed from a cotton rat. Ticks were preserved in 70% ethanol or frozen at –80ºC until evaluation.

Most individual ticks were minced with a sterile scalpel blade, and DNA was extracted by using a QIAamp Mini Kit (QIAGEN, Inc., Valencia, CA, USA). Others were minced or crushed after freezing in liquid nitrogen, and DNA was extracted by using an IsoQuick nucleic acid extraction kit (ORCA Research, Bothell, WA, USA). DNA extracts were evaluated by using nested or heminested PCR assays designed to amplify a segment of the *rompA* gene. For the primary stage of each assay, 5 µL of extract and primers 190–70 and 190–701 (*9*) were used. For the nested reaction, 2 µL of completed primary reaction was used as template with primers 190-FN1 (5′-AAG CAA TAC AAC AAG GTC-3′) and 190-RN1 (5′-TGA CAG TTA TTA TAC CTC-3′); for the heminested reaction, primers 190–FN1 and 190–701 were used. All reactions were prepared by using a High Fidelity PCR Master Kit (Roche Diagnostics, Indianapolis, IN, USA) with final primer concentrations of 300 nmol in a total volume of 50 μL. Thermalcycler parameters for the primary stage consisted of an initial denaturation period of 2 min at 94^o^C, followed by 40 cycles of 15 s at 94^o^C, 30 s at 60^o^C, 45 s at 72^o^C, and a 5-min extension period at 72^o^C. For the nested and heminested stages, the annealing temperature was changed to 55^o^C and the number of cycles was reduced to 30.

PCR products (10 μL) were separated by electrophoresis in 2% agarose gels containing ethidium bromide. For each positive reaction, the remaining 40 μL was subjected to gel electrophoresis, and products of the appropriate size were excised. DNA was purified from the gel fragments by using the QIAquick Gel Extraction Kit (QIAGEN). Purified PCR products were sequenced using the PCR primers and the GenomeLab DTCS Quick Start Kit (Beckman Coulter, Fullerton, CA, USA). For some products, additional sequencing primers 190-SF3 (5′-GGT ACT ACT CCC GTA GGT C-3′) and 190-SR2 (5′-CCG GCA GTA AKA GTA ACA G-3′) were used to obtain complete sequences for both strands. Sequences were detected by using a Beckman CEQ 8000 automated sequencer. Sequence similarities were determined by using the BLAST program (version 2.0, National Center for Biotechnology Information, www.ncbi.nlm.nih.gov/blast). Sequence-length reaction products (excluding primers) were 590 bp for primary, 559 bp for heminested, and 540 bp for nested.

DNA of *R. parkeri* was amplified from 21 (11.5%) of 182 male and female adult *A. maculatum* ticks collected in Georgia (11/64), Florida (7/89), Kentucky (1/1), Mississippi (1/24), and South Carolina (1/4), and from 1 engorged nymph collected in Oklahoma (Table). A unique *rompA* sequence (GenBank accession no. EF372578) amplified from 5 adult female ticks collected in Florida, Georgia, and Mississippi showed closest homology (≈94%) to several other *rompA* sequences (GenBank accession nos. EF063690, AY093696, AF120021, and DQ365801). PCR amplification of a 208-bp segment of the rickettsial 17-kDa antigen gene (*10*) from these same 5 ticks (GenBank accession no. EF372579) showed 100% homology with the corresponding sequences of *Rickettsia* sp. Arahna (AY360215), *R. montanensis* (DQ402377), *Rickettsia* sp. Hf332 (AB114804), *Rickettsia* sp. Is-1 (DQ344620), and “R. gravesii” (DQ269436).

**Table:** Gulf Coast ticks (*Amblyomma maculatum*) infected with spotted fever group rickettsiae, United States, 1996–2005

*Rickettsia* sp. identified	State	County	Total ticks positive/total ticks tested*	Year collected	Stage, sex†	Source
*Rickettsia parkeri*	Florida	Duval	1/9	1999	Adult, F	Vegetation
		Franklin	3/25	2004	Adults, M, F	Vegetation
			3/38	2005	Adults, M, F	Vegetation
	Georgia	Bulloch	2/20	1999	Adults, M, F	Vegetation
			5/16	2003	Adults, M, F	Vegetation
			3/24	2005	Adults, M, F	Vegetation
		McIntosh	1/4	2005	Adult, F	Vegetation
	Kentucky	Montgomery	1/1	2003	Adult, M	Human
	Mississippi	Copiah	1/9	2002	Adult, M	Vegetation
	Oklahoma	Pittsburgh	1/1	1997	Nymph‡	*Sigmodon hispidis*
	South Carolina	Anderson	1/4	2005	Adult, M	*Canis latrans*
Noncharacterized *Rickettsia* sp.§	Florida	Nassau	1/13	1996	Adult, F	Vegetation
		Franklin	1/38	2005	Adult, F	Vegetation
	Mississippi	Copiah	1/9	2002	Adult, F	Vegetation
	Georgia	Bulloch	1/20	1999	Adult, F	Vegetation
			1/24	2005	Adult, F	Vegetation

­­­­­­­­­­­­­­*Some specimens obtained from a given county during a given year were not collected synchronously. †Identified by PCR; nonengorged specimens unless otherwise specified. ‡Engorged specimen. §Based on a unique *rompA* sequence.

Precise estimates of infection prevalence could not be assessed from these data because most of the ticks evaluated in this study were collected as relatively small sample sizes or were collected in a discontinuous manner as multiple samples from the same sites over several weeks or months in a given year. However, some collections were flagged synchronously at a single location, including those in Copiah County, Mississippi, during July 2002 (n = 9) and in Franklin County, Florida, during July 2004 (n = 25) and July 2005 (n = 27). Infection prevalence for each of these 3 collections was 11%–12%, which suggests that *R. parkeri* may be a relatively common inhabitant of some populations of Gulf Coast ticks*.* By comparison, the estimated prevalence of infection of tick vectors with *R. rickettsii*, the etiologic agent of Rocky Mountain spotted fever (RMSF), is typically much lower than that found by surveys elsewhere, which identified *R. rickettsii* in only 0.05%–1.3% of the collected specimens: 3,705 *Dermacentor andersoni* ticks from canyons bordering the Bitterroot Valley of Montana; 2,123 and 310 *D. variabilis* ticks from RMSF-endemic areas of North Carolina and Ohio, respectively; and 669 *A. aureolatum* ticks from São Paulo, Brazil (*11**–**14*)*.*

## Conclusions

*R. parkeri* has been isolated in culture from Gulf Coast ticks collected in Alabama, Florida, Georgia, Mississippi, and Texas ([*2,7,8*]*,* C. Paddock, unpub. data). These results, combined with data from the present study, suggest that in the United States *R. parkeri* can be found anywhere that *A.* *maculatum* ticks are found. In this context, persons exposed to habitats in any region infested by Gulf Coast ticks are potentially vulnerable to infection with *R. parkeri*. A previously undescribed *rompA* sequence, identified in a few ticks collected during this survey, may represent a novel species of spotted fever group rickettsiae associated with the Gulf Coast tick. Attempts to isolate and characterize this species are in progress. Collectively, these findings suggest that the role of *A. maculatum* in the ecology of various spotted fever group rickettsiae deserves further attention. These results and the recent discovery of *R. parkeri* in *A. triste* ticks in Uruguay (*15*) indicate that much remains to be learned about *R. parkeri* and other rickettsiae of human-biting ticks in the Western Hemisphere and their relative contributions to the epidemiology of New World spotted fevers.
